# Clinical outcomes in patients with node-negative breast cancer treated based on the recurrence score results: evidence from a large prospectively designed registry

**DOI:** 10.1038/s41523-017-0034-6

**Published:** 2017-09-08

**Authors:** Salomon M. Stemmer, Mariana Steiner, Shulamith Rizel, Lior Soussan-Gutman, Noa Ben-Baruch, Avital Bareket-Samish, David B. Geffen, Bella Nisenbaum, Kevin Isaacs, Georgeta Fried, Ora Rosengarten, Beatrice Uziely, Christer Svedman, Debbie McCullough, Tara Maddala, Shmuel H. Klang, Jamal Zidan, Larisa Ryvo, Bella Kaufman, Ella Evron, Natalya Karminsky, Hadassah Goldberg, Steven Shak, Nicky Liebermann

**Affiliations:** 1 Davidoff Center, Rabin Medical Center, 39 Jabotinski St., Petah Tikva, 49414 Israel; 2 Sackler Faculty of Medicine, Tel Aviv University, Tel Aviv, Israel; 3 Lin Medical Center, Haifa, Israel; 4 Oncotest Division, Teva Pharmaceutical Industries, Ltd., Shoham, Israel; 5 Oncology Dept., Kaplan Medical Center, Rehovot, Israel; 6 BioInsight Ltd, Zichron Yaakov, Israel; 7 Department of Oncology, Soroka University Medical Center and the Faculty of Health Sciences, Ben-Gurion University of the Negev, Beer Sheva, Israel; 8 Oncology Dept., Meir Medical Center, Kfar Saba, Israel; 9 Oncology Dept., Ha’emek Medical Center, Afula, Israel; 10 Oncology Dept., Rambam Health Care Campus, Haifa, Israel; 11 Oncology Institute, Shaare Zedek Medical Center, Jerusalem, Israel; 12 Sharett Institute of Oncology, Hadassah-Hebrew University Medical Center, Jerusalem, Israel; 13 Genomic Health Inc., Redwood City, CA USA; 14 Community Division, Clalit Health Services, Tel Aviv, Israel; 15 The Hebrew University, Faculty of Medicine, School of Pharmacy, Jerusalem, Israel; 16 Oncology Dept., Ziv Medical Center, Safed, Israel; 17 Faculty of Medicine, Bar Ilan University, Safed, Israel; 18 Oncology Dept., Tel-Aviv Sourasky Medical center, Tel Aviv, Israel; 19 Oncology Dept., Sheba Medical Center, Ramat Gan, Israel; 20 Oncology Dept., Assaf Harofeh Medical Center, Zerifin, Israel; 21 Oncology Dept., Wolfson Medical Center, Holon, Israel; 22 Galilee Medical Center, Nahariya, Israel

## Abstract

The 21-gene Recurrence Score® (RS) assay is a validated prognostic/predictive tool in ER + early-stage breast cancer. However, clinical outcome data from prospective studies in RS ≥ 11 patients are lacking, as are relevant real-life clinical practice data. In this retrospective analysis of a prospectively designed registry, we evaluated treatments/clinical outcomes in patients undergoing RS-testing through Clalit Health Services. The analysis included N0 ER + HER2-negative breast cancer patients who were RS-tested from 1/2006 through 12/2010. Medical records were reviewed to verify treatments/recurrences/survival. The cohort included 1801 patients (median follow-up, 6.2 years). Median age was 60 years, 50.4% were grade 2 and 81.1% had invasive ductal carcinoma; 48.9% had RS < 18, 40.7% RS 18–30, and 10.4% RS ≥ 31, with chemotherapy use of 1.4, 23.7, and 87.2%, respectively. The 5-year Kaplan–Meier estimates for distant recurrence were 0.8, 3.0, and 8.6%, for patients with RS < 18, RS 18–30 and RS ≥ 31, respectively; the corresponding 5-year Kaplan–Meier estimates for breast cancer death were 0.0, 0.9, and 6.2%. Chemotherapy-untreated patients with RS < 11 (*n* = 304) and 11–25 (*n* = 1037) (TAILORx categorizatio*n*) had 5-year Kaplan–Meier estimates for distant recurrence risk/breast cancer death of 1.0%/0.0% and 1.3%/0.4%, respectively. Our results extend those of the prospective TAILORx trial: the 5-year Kaplan–Meier estimates for distant recurrence and breast cancer death rate for the RS < 18 patients were very low supporting the use of endocrine therapy alone. Furthermore, in chemotherapy-untreated patients with RS 11–25 (where TAILORx patients were randomized to chemoendocrine or endocrine therapy alone), 5-year distant recurrence rates were also very low, suggesting that chemotherapy would not have conferred clinically meaningful benefit.

## Genetic testing: Diagnostic shows which node-negative patients need chemo

Patients with early breast cancer that hasn’t spread to lymph nodes can likely forgo chemotherapy if they score under 25 on Oncotype DX. That’s the finding of a retrospective analysis led by Salomon Stemmer from Rabin Medical Center in Petah Tikvah, Israel, that looked at 1801 women with node-negative, ER-positive, HER2-negative disease who received the diagnostic test, which measures the expression levels of 21 genes within tumor cells. Rates of disease recurrence and death were low for patients who received only anti-hormone treatment and had low-to-intermediate Oncotype DX results, suggesting no need for additional chemotherapy (which carries an appreciable risk of toxicity). Previously, a prospective US study called TAILORx established that women with scores under 11 could be spared chemotherapy. The Israeli trial validates and extends the results to include women with scores up to 25.

## Introduction

The 21-gene Recurrence Score® (RS) assay (Oncotype DX®, Genomic Health Inc., Redwood City, CA) is used to guide treatment decisions in estrogen receptor (ER) + early-stage breast cancer (BC) for more than a decade. The assay was initially validated as a prognostic/predictive tool in multiple prospectively-designed studies using archival specimens of clinical trials with long-term follow-up.^1–6^ In the first validation study,^1^ RS risk groups (low, <18; intermediate, 18–30; high, ≥ 31) were defined and validated. The assay is now included in major international guidelines.^7–10^

Prospective outcome data from patients treated based on their RS results are currently limited to findings from 2 phase 3 trials; TAILORx and West German Study Group (WSG) PlanB. TAILORx is a non-inferiority trial comparing endocrine treatment alone to chemoendocrine treatment in patients with node-negative, hormone receptor (HR)+, human epidermal growth factor receptor 2 (HER2)−negative BC and RS 11–25. Patients with RS > 25 received chemoendocrine therapy, and patients with RS < 11 received endocrine therapy alone. Recently published TAILORx findings reported only on the RS < 11 patients (*n* = 1626), and demonstrated very low recurrence rates (rate of freedom from distant recurrence at 5 years: 99.3%; overall survival at 5 years, 98.0%).^11^ Prospective outcome data have also been presented from the WSG PlanB trial, where patients with HR + HER2-negative disease with node-positive or high risk node-negative early BC and RS ≤ 11 were recommended to omit adjuvant chemotherapy. The 3-year disease-free survival for the 348 RS ≤ 11 chemotherapy-untreated patients was 98%.^12^

The TAILORx data from patients with RS 11–25 randomized to chemoendocrine or endocrine therapy alone has yet to be presented. While awaiting these results to clarify whether chemotherapy benefits intermediate risk patients, prospective data on recurrence rates in patients with RS ≥ 11 treated with endocrine therapy alone are lacking, as are data from real-life clinical practice where treatment decisions incorporated the RS.

The RS assay became commercially available in 2004. Clalit Health Services (CHS), the largest health maintenance organization (HMO) in Israel, approved assay reimbursement for node-negative ER + patients in 1/2006 and extended its policy in 1/2008 to include node-positive (up to 3 positive axillary lymph nodes including micrometastases) patients. Since its introduction in Israel, approximately 11,600 patients have been RS-tested including approximately 7300 CHS members. Here, we report the first outcome results of a real-life contemporary practice CHS registry analysis where all patients were RS-tested > 5 years ago. The relationship between the RS, adjuvant treatments received, and clinical outcomes across the entire range of RS results is reported. Notably, this analysis complements another analysis focusing on node-positive patients who were RS-tested through CHS.^13^

## Results

### Patient characteristics

**Fig. 1:**
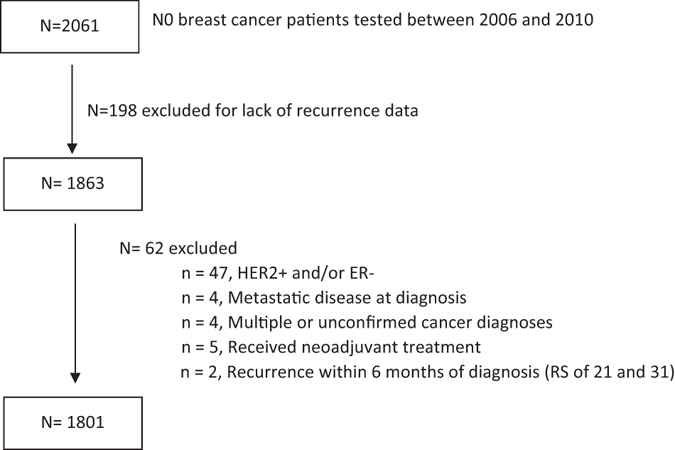
Patient disposition

Patient disposition is described in Fig. 1. Between 1/2006 and 12/2010, 2061 CHS members with N0 BC were RS-tested. Recurrence data were not available for 198 patients (<10%) for various reasons (patients switched to another HMO, relocated to another country, etc). In addition, 62 patients were excluded for other reasons (Fig. 1). The final cohort included 1801 patients. The median follow-up was 6.2 years.

Table 1 shows patient/tumor characteristics for the cohort. The vast majority (99%) were female. Median age was 60 (interquartile-range: 52–67) years, 83.6% were ≥ 50 years. Approximately half (50.4%) had grade 2 tumors, 77.5% had tumors ≤ 2 cm in size, and 81.1% had invasive ductal carcinoma.

**Table 1:** Baseline patient and tumor characteristics

	All patients	RS < 11	RS: 11-<18	RS: 18–25	RS: 26–30	RS ≥ 31
	*N* = 1801	*n* = 304	*n* = 576	*n* = 562	*n* = 171	*n* = 188
Gender, *n* (%)						
Female	1787 (99)	301 (99)	573 (99)	558 (99)	169 (99)	186 (99)
Male	14 (<1)	3 (1)	3 (1)	4 (1)	2 (1)	2 (1)
Age						
Median (interquartile range), years	60 (52–67)	63 (56–71)	59 (51–66)	59 (53–66)	60 (54–66)	59 (50–66)
Mean (SD), years	59.4 (10)	62.9 (10)	58.7 (10)	58.9 (9)	59.7 (10)	57.6 (12)
Age category, *n* (%)						
<40 years	47 (2.6)	3 (1)	7 (1)	15 (3)	4 (2)	18 (10)
40–49 years	248 (13.8)	31 (10)	96 (17)	74 (13)	20 (12)	27 (14)
50–59 years	580 (32.2)	67 (22)	204 (35)	199 (35)	59 (35)	51 (27)
60–69 years	604 (33.5)	113 (37)	178 (31)	198 (35)	59 (35)	56 (30)
70–79 years	295 (16.4)	81 (27)	80 (14)	72 (13)	27 (16)	35 (19)
≥80 years	27 (1.5)	9 (3)	11 (2)	4 (1)	2 (1)	1 (0)
Tumor size in the greatest dimension						
Median (interquartile range), cm	1.5 (1.1, 2.0)	1.5 (1.1–2.0)	1.5 (1.0–2.0)	1.5 (1.0–2.0)	1.5 (1.2–2.0)	1.8 (1.5–2.5)
Mean (SD), cm	1.7 (0.8)	1.6 (0.8)	1.6 (0.8)	1.6 (0.8)	1.7 (0.8)	2.0 (0.9)
Tumor size category, *n* (%)						
≤1 cm	400 (22.2)	63 (21)	143 (25)	140 (25)	32 (19)	22 (12)
>1–2 cm	996 (55.3)	181 (60)	320 (56)	302 (54)	103 (60)	90 (48)
>2–3 cm	313 (17.4)	48 (16)	84 (15)	94 (17)	28 (16)	59 (31)
>3	80 (4.4)	11 (4)	24 (4)	23 (4)	7 (4)	15 (8)
Unknown	12 (0.7)	1 (0)	5 (1)	3 (0)	1 (1)	2 (1)
Tumor grade category, *n* (%)						
Grade 1	258 (14.3)	61 (20)	109 (19)	73 (13)	10 (6)	5 (3)
Grade 2	907 (50.4)	173 (57)	291 (51)	283 (51)	80 (47)	80 (43)
Grade 3	297 (16.5)	15 (5)	56 (10)	93 (17)	48 (28)	85 (45)
Not applicable/Unknown^a^	339 (18.8)	55 (18)	120 (21)	113 (20)	33 (19)	18 (10)
Tumor grade and size, *n* (%)						
Grade 1 and tumor size ≤1 cm	90 (4.9)	15 (5)	44 (8)	28 (5)	3 (2)	0 (0)
Histology, *n* (%)						
IDC	1461 (81.1)	274 (81)	451 (78)	454 (81)	139 (81)	170 (90)
ILC	213 (11.8)	23 (8)	80 (14)	78 (14)	23 (14)	9 (5)
Mucinous/colloid	53 (2.9)	14 (4)	20 (4)	13 (2)	5 (3)	1 (1)
Papillary	21 (1.2)	10 (3)	6 (1)	3 (1)	0 (0)	2 (1)
Other	53 (2.9)	10 (3)	19 (3)	14 (2)	4 (3)	6 (3)

*IDC* invasive ductal carcinoma, *ILC* invasive lobular carcinoma, *RS* recurrence score

^a^ 59.8% of unknown tumor grade are ILC

### RS distribution and patient characteristics within RS subgroups

Of the 1801 patients, 880 (48.9%) had RS < 18, 733 (40.7%) had RS 18–30, and 188 (10.4%) had RS ≥ 31. A wide RS distribution was observed within each level of clinicopathological characteristic including age, tumor size, and tumor grade (Supplementary Fig. S1 a–c). Patient characteristics in RS subgroups (<11, 11-<18, 18–25, 26–30, and ≥31) seemed similar with respect to age and tumor size (Table 1). The lower RS groups had higher proportion of grade 1 tumors and lower proportion of grade 3 tumors compared with the higher RS groups. Patients with very low risk by clinicopathological characteristics (grade 1 and tumor size ≤1 cm) were observed in all RS subgroups except for the RS ≥ 31 group (Table 1).

### Adjuvant chemotherapy treatment

Chemotherapy use was consistent with the RS with 1.4% (12/880), 23.7% (174/733), and 87.2% (164/188) receiving adjuvant chemotherapy in patients with RS < 18, 18–30, and ≥31, respectively. The overall chemotherapy rate was 19.4%. Within the RS 18–30 group, chemotherapy use increased with increasing RS results (Supplementary Fig. S2). Chemotherapy-treated and untreated patients were overall similar with respect to clinicopathological characteristics (Supplementary Table S1).

### Distant recurrence rates

**Fig. 2:**
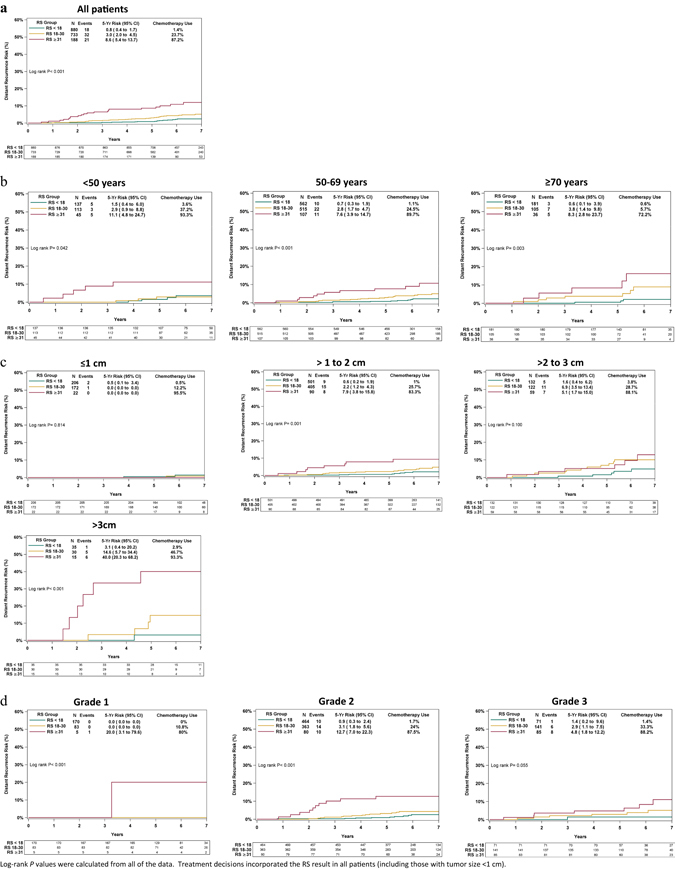
Kaplan–Meier distant recurrence curves for the entire cohort and by clinicopathologic characteristics. Rates of distant recurrence for the entire cohort (**a**), by age (**b**), tumor size (**c**), and tumor grade (**d**). For each RS category, the percentage of patients receiving chemotherapy is indicated. The *box* under each graph presents the number of patients at risk at each time point

With a median follow-up of 6.2 years, 71 distant recurrences were documented; 18/880, 32/733, and 21/188 in patients with RS results <18, 18–30, and ≥31, respectively. Kaplan–Meier (KM) estimates for distant recurrence within 5 years differed significantly (*P* < 0.001) between the RS groups with rates of 0.8% (95% confidence interval [CI], 0.4–1.7%) in the RS < 18, 3.0% (95% CI, 2.0–4.5%) in the RS 18–30, and 8.6% (95% CI, 5.4–13.7%) in the RS ≥ 31 group (Fig. 2a). Subgroup analyses by age, tumor size, and tumor grade showed that the difference in distant recurrence risk between the RS groups was statistically significant in all evaluated clinicopathological subgroups, and that RS < 18 patients had low distant recurrence risk regardless of age, tumor size and tumor grade (Fig. 2b–d). Within the RS ≥ 18 group (where the recurrence risk was higher across clinicopathological levels), larger tumor size (>3 cm) seemed to be associated with higher distant recurrence risk (Fig. 2d).

**Fig. 3:**
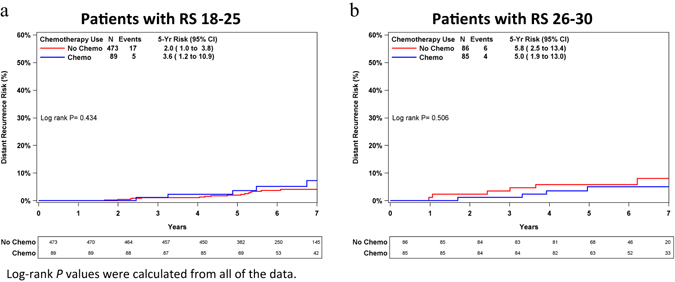
Kaplan–Meier distant recurrence curves in patients with RS 18–25 and 26–30 by chemotherapy use. **a** Patients with RS 18–25. **b** Patients with RS 26–30. The *box* under each graph presents the number of patients at risk at each time point

We divided the RS 18–30 group into two subgroups (using the TAILORx RS cutoff value of 25): 18–25 and 26–30. KM estimates for distant recurrence risk by chemotherapy use for each subgroup are presented in Fig. 3. It should be emphasized that results are to be interpreted cautiously, as patients were not randomized to treatment (of the RS 18–30 patients, 174 were chemotherapy-treated and 559 were untreated) and there is likely a selection bias for choice of therapy. Within each of these subgroups, the KM curves were similar and there was no statistically significant difference in 5-year distant recurrence risk between treated and untreated patients (*P ≥ *0.4). As sensitivity analysis, propensity score (PS) adjustment was utilized within three subgroups: N0 patients with RS 18–25, N0 patients with RS 26–30, and all patients with RS 18–30. Age, tumor size, and grade were used for calculating PS. The PS-adjusted models produced similar results to the unadjusted models. In all cases, there were no significant differences in time to distant recurrence between chemotherapy-treated and untreated patients.

**Fig. 4:**
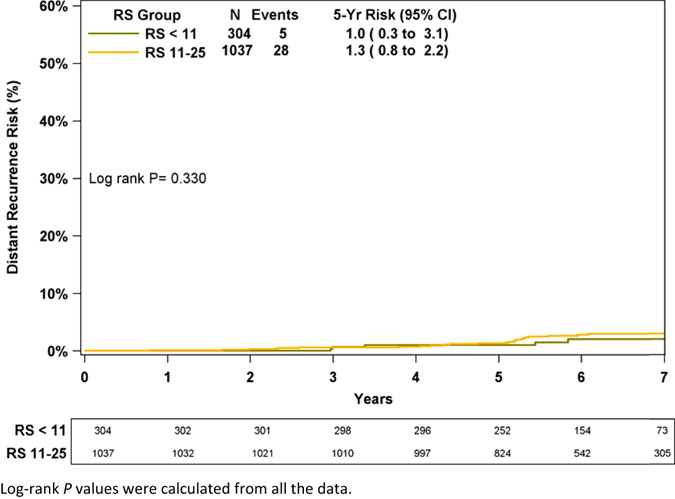
Kaplan–Meier distant recurrence curve in chemotherapy-untreated patients by TAILORx RS categories (RS < 11, RS 11–25). The *box* under the graph presents the number of patients at risk at each time point

Additionally, the TAILORx categorization (<11, 11–25)^11^ was used for distant recurrence risk analysis in N0 chemotherapy-untreated patients (93% of patients with RS ≤ 25) (Fig. 4). The KM estimate for distant recurrence risk at 5 years was very low in both the RS < 11 and 11–25 categories (1.0% [95% CI, 0.3–3.1%] and 1.3% [95% CI, 0.8–2.2%], respectively) and consistent with the TAILORx results for RS < 11.^11^

### Breast cancer death rates

**Fig. 5:**
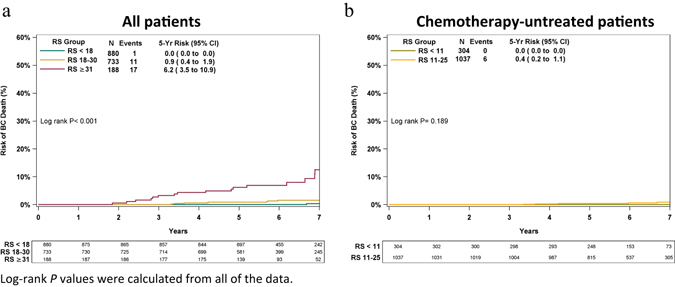
Kaplan–Meier breast cancer death. Risk of breast cancer death by RS risk groups (<18, 18–30, ≥31) in all patients (chemotherapy treated and untreated) **a**, and in chemotherapy-untreated patients by TAILORx RS categorization (RS < 11, RS 11–25) **b**. The *box* under each graph presents the number of patients at risk at each time point

Twenty-nine BC deaths were documented (1/880, 11/733, and 17/188 in the RS < 18, RS 18–30, and RS ≥ 31 groups, respectively). KM estimates for the risk of BC death within 5 years differed significantly (*P* < 0.001) between the RS groups (Fig. 5a); this risk was 0.0% (no deaths reported in the first 5 years) in RS < 18, 0.9% (95% CI, 0.4–1.9%) in RS 18–30, and 6.2% (95% CI, 3.5–10.9%) in the RS ≥ 31 group. In an analysis using TAILORx categorization in N0 chemotherapy-untreated patients (Fig. 5b), the KM estimates for 5-year BC death risk was very low in both the RS < 11 and RS 11–25 TAILORx categories (0.0% [95% CI, 0.0–0.0%] and 0.4% [95% CI, 0.2–1.1%], respectively).

### Multivariable analysis

A multivariable regression analysis was performed on the entire cohort and included the RS group (18–30 vs. <18, ≥31 vs. <18), age (per year), size (≤2 vs. >2 cm), and histologic grade (2 vs. 3, 1 vs. 3). It showed that both the RS group and tumor size had a significant association with distant recurrence risk. The hazard ratio [HR] for RS 18–30 vs. RS < 18 was 2.0 (95% CI, 0.97–4.3), and for RS ≥ 31 vs. RS < 18, 5.8 (95% CI, 2.6–12.9); *P* < 0.0001. For tumor grade 1 vs. 3, it was 0.2 (95% CI, 0.02–1.5), and for grade 2 vs. 3, 1.3 (95% CI, 0.7–2.6); *P* = 0.118; for size, the HR was 2.3 (95% CI, 1.3–4.0), *P* = 0.006 for >2 vs. ≤2 cm (Supplementary Table S2).

## Discussion

These are the first clinical outcome data from an analysis of a large prospectively designed registry investigating patients across the full RS range, where the assay has been incorporated into real-life clinical practice. We found that chemotherapy use was consistent with the RS, and that clinical outcomes were very good overall and excellent in chemotherapy-untreated patients with RS ≤ 25.

Our results are consistent with the original validation studies,^2, 14^ and complement recent findings from TAILORx, the SEER registry analysis, and WSG PlanB.^11, 12, 15^ The 5-year KM recurrence rate in patients with RS < 11 treated with endocrine alone was very similar in our analysis and TAILORx (1.0 and 0.7%, respectively) allowing us to assume that our population is similar to that in TAILORx. Furthermore, the 5-year KM recurrence rate for the *entire* RS < 18 group (which in our cohort included 1.4% chemotherapy-treated patients) was 0.8%, which is comparable to that reported in TAILORx for RS < 11 (where all patients were chemotherapy-untreated).^11^ Notably, our analysis included all N0 RS-tested patients regardless of tumor size/grade, whereas TAILORx included node-negative patients with a primary tumor size of 1.1–5.0 cm in the longest dimension (any grade) or those with tumors 0.6–1.0 cm in the longest dimensions and grade 2 or 3.^11^ Despite the differences in inclusion/exclusion criteria, the pathological characteristics of the patients in our analysis and in TAILORx are similar with respect to median tumor size and age. Our cohort seems to have lower proportion of grade 1 tumors compared with TAILORx. Approximately 12% of our chemotherapy-untreated patients would not have been eligible for TAILORx. Our findings also complement the SEER analysis, which was limited to survival data, and demonstrated excellent (99.6%) 5-year BC-specific survival in >21,000 N0 HR + HER2-negative BC patients with RS < 18.^15^ Our results are also consistent with those reported for the WSG PlanB analysis where the patient population had overall higher risk of recurrence based on clinicopathological characteristics than in our patient population.^12^ The PlanB analysis included HR + HER2-negative node-positive or high-risk (T2, grade 2 or 3, high uPA/PAI-1, or <35 years of age) node-negative patients. PlanB patients with RS ≤ 11 who did not receive adjuvant chemotherapy (*n* = 348) had 3-year disease-free survival of 98%, whereas those with RS 12–25 and those with RS > 25 (all of whom received chemotherapy) had 3-year disease-free survival of 98 and 92%, respectively.^12^

Prospective–retrospective validation studies showed that RS < 18 patients gain minimal, if any, benefit from chemotherapy, whereas for those with RS 18–30, some chemotherapy benefit cannot be ruled out.^3, 4^ Our registry analysis supports using endocrine therapy alone in RS < 18 patients. It is also consistent with the decision by many physicians to spare chemotherapy in certain patients with RS ≤ 25, as patients selected for endocrine therapy alone in our cohort had excellent clinical outcomes without adjuvant chemotherapy: 5-year KM-estimate for distant recurrence rate of 1.0% for patients with RS < 11 and 1.3% for patients with RS 11–25. Since the distant recurrence rate without adjuvant chemotherapy is so low in this population, the absolute benefit of chemotherapy is expected to be very small, and to be outweighed by treatment-related toxicity and mortality. In addition, our analysis of chemotherapy-treated vs. untreated patients showing no significant difference in recurrence risk between these groups supports treating these patients with endocrine therapy alone. In the group of patients with RS 26–30, no evidence of chemotherapy benefit was observed although the number of patients and events in this group was small; in a post hoc analysis of statistical power for detecting a difference in outcome by treatment, we calculated less than 30% power in both the RS 18–25 and RS 26–30 cohorts. Notably, our registry analysis is impacted by selection bias as patients were not randomized to chemoendocrine vs. endocrine therapy alone. For patients with RS 18–30, measured/unmeasured prognostic parameters associated with higher risk maybe more common in those who were recommended chemotherapy. The results from the randomized arms in TAILORx will provide final evidence on whether endocrine therapy alone is non-inferior to chemoendocrine therapy in this patient population.

Although some subgroups are relatively small, our findings suggest that RS ≥ 18, patients with larger tumors have worse outcomes; however, we cannot predict whether these patients will benefit from adjuvant treatment with the currently used chemotherapeutic agents.

Our data, showing that oncologists in Israel treat BC based on the RS results, are consistent with decision impact studies conducted worldwide (including in Israel).^16–23^ Within the RS 18–30 group, we found increased chemotherapy use with higher RS results, suggesting that clinicians do not view this group as a uniform entity.

Notably, other genomic assays are also commercially available and serve as prognostic tools in early BC. These include MammaPrint® (Agendia BV, Amsterdam, The Netherlands), Prosigna® (PAM50; NanoString Technologies Inc, Seattle WA), and EndoPredict® (Myriad Genetics Inc, Salt Lake City, UT). These assays differ from each other and from the RS assay in the technological platforms used, the specific genes included in the assay, and the patient populations used for their development/validation.^24–28^

There are health economic (HE) implications associated with the cost of the tests, from sparing chemotherapy among patients who would not benefit from it, thereby decreasing acute/chronic treatment-related morbidity, as well as from using chemotherapy in patients who would benefit from it (i.e., preventing recurrences). The first 313 RS assays reimbursed by CHS were included in HE analysis, which reported cost effectiveness.^29^ A formal cost-effectiveness evaluation of using the RS assay in Israel is warranted and planned. Notably, our distribution analyses demonstrated that overall, estimating the RS from clinicopathological characteristics is not feasible as the RS distribution was wide in all evaluated subgroups, except for patients with grade 1 disease and tumors ≤1 cm where the proportion of patients with RS > 25 was low and the baseline risk to which the RS adds prognostic information is also low.

This is the first real-life large registry analysis that represents clinical practice on a national level; no exclusion criteria with respect to gender, age, location, socioeconomic status, or comorbidities were applied and patients were treated in CHS-affiliated centers and government hospitals throughout Israel. Nonetheless, the analysis does have some limitations. It was not randomized and patients were not treated uniformly (with respect to chemotherapy/endocrine therapy regimens), and there may have been selection bias in who was tested and in those not receiving chemotherapy. We believe that the former was minimal as >80% of eligible CHS patients are tested. Also, although the sample size was approximately 1800 patients, the event rate was very low, and therefore drawing more definitive conclusions for smaller subgroups is not possible. The SEER analysis may provide robust data in subgroups with less common clinicopathological characteristics. Also, median follow-up of 6 years is relatively short for N0 ER + patients; thus, our findings are limited to ‘early’ distant recurrences, but this is also the timeframe when chemotherapy benefit would be expected. Longer-term analysis (median of 10 years) is warranted and planned. The TAILORx randomized arms are yet to be reported as the number of events has been low (consistent with our findings).

In conclusion, this is the first analysis of a prospectively designed registry evaluating clinical outcomes in N0 ER + HER2-negative early BC patients for whom the RS was used in real-life clinical practice. It extends the remarkable results from the TAILORx-defined cohort (RS < 11) to include patients with RS < 18, as in both studies, the 5-year risk of distant recurrence was very low (≤1%). For selected patients with RS ≤ 25, the data suggest that endocrine therapy alone may suffice.

## Methods

### Patient population

Collecting clinical outcome data from all CHS RS-tested patients was planned by CHS, in concert with assay reimbursement approval. This retrospective analysis of the prospectively designed CHS registry investigated the relationship between the RS, adjuvant treatments received, and distant recurrence/survival in patients with ER + HER2-negative N0 BC in real-life clinical practice. The analysis included all N0 CHS members who were RS-tested between 1/2006 through 12/2010 to allow for ≥5 years of follow-up. Patients who were ER-negative by immunohistochemistry (IHC; cutoff: 0.5) and reverse transcription polymerase chain reaction (RT-PCR), which was performed as part of the 21-gene assay (cutoff: 6.5 units) were excluded. Also, those who were HER2 + by IHC (cutoff: 3), fluorescence in situ hybridization (ratio cutoff: 2.2), or RT-PCR performed as part of the 21-gene assay (cutoff: 10.7 units), and patients who received trastuzumab adjuvantly were also excluded. Patients receiving neoadjuvant treatment, those with metastatic disease at the time of testing, and patients recurring within 6 months of testing (as it was assumed that they were metastatic at diagnosis), as well as those with another malignancy for which they were treated within 6 months of testing were also excluded.

This analysis was approved by the institutional review boards of the CHS Community Division and participating medical centers and was granted a waiver for obtaining patient consent.

### Data source

RS results and patient/tumor characteristics were extracted from the Teva Pharmaceutical Industries Oncotest database. Clinical information (treatments, death status) was extracted from CHS claims arm. Medical records were used to verify treatments received and recurrence status (reviewers were unaware of the RS results).

### Statistical analysis

The statistical analysis plan was pre-specified. Descriptive statistics were used to summarize clinicopathological characteristics. The primary endpoint was 5-year KM distant recurrence estimates and 95% CI by RS risk categories^1^ (<18, 18–30, ≥31). Patients without recurrence were censored at the time of last follow-up (later of last follow-up or date that medical records were reviewed) or at time of death (due to any cause). A secondary endpoint was 5-year KM BC death estimates and 95% CI. Patients with metastatic disease at the time of death were considered events; patients were censored at the time of last follow-up or death from other causes, and recurrences were ignored for purposes of this endpoint. Analysis was also performed on intermediate risk^1^ subgroups (18–25, 25–30) and using TAILORx^11^ RS categorization (<11 and 11–25). The log-rank test was used to compare distant recurrence rates across RS groups. Cox proportional hazards regression models were used to evaluate the association of RS group, age, size, and grade with distant recurrence. Univariate analysis identified a set of prognostic baseline factors, and a full multivariable model was fit. Non-significant covariates were removed from the final model. For sensitivity analysis, two sets of prognostic models were fit: one assessing fit with the three-category RS group, the other with continuous RS result. Proportional hazards assumptions were assessed and met in the final model. As sensitivity analysis, PS adjustments were performed to account for lack of randomization. SAS 9.4 (SAS Institute Inc., Cary, NC) was used for the analysis. *P* < 0.05 was considered statistically significant.

### Data availability

All relevant data are available upon request from the corresponding author.

## Electronic supplementary material

Supplementary information
