# Supplementary material for: Clinical outcomes in patients with node-negative breast cancer treated based on the recurrence score results: evidence from a large prospectively designed registry
Source: NPJ Breast Cancer. 2017 Sep 8;3:33. doi: 10.1038/s41523-017-0034-6 (PMC5591181; doi:10.1038/s41523-017-0034-6)
Supplement: Supplementary file 1 — Supplementary information [file 41523_2017_34_MOESM1_ESM.pdf]

SUPPLEMENTARY INFORMATION

Table S1. Baseline patient and tumor characteristics by treatment.

	All patients *N* = 1801	Patients not treated with adjuvant chemotherapy *n* =1451	Adjuvant chemotherapy-treated patients *n* = 350
Gender, *n* (%)			
Female	1787 (99)	1440 (99)	347 (99)
Male	14 (1)	11 (1)	3 (1)
Age			
Median (interquartile range), years	60 (52-67)	61 (53-68)	56 (49-63)
Mean (SD), years	59.4 (10)	60 (10)	55.8 (10)
Age category, *n* (%)			
<40 years	47 (2.6)	18 (1)	29 (8)
40-49 years	248 (13.8)	188 (13)	60 (17)
50-59 years	580 (31.4)	456 (31)	124 (35)
60-69 years	604 (33.5)	500 (34)	104 (30)
70-79 years	295 (16.4)	262 (18)	33 (9)
≥80 years	27 (1.5)	27 (2)	0 (0)
Tumor size in the greatest dimension			
Median (interquartile range), cm	1.5 (1.2, 2.0)	1.5 (1.1-2.0)	1.7 (1.3-2.5)
Mean (SD), cm	1.7 (0.8)	1.6 (0.8)	1.9 (1.0)
Tumor size category, *n* (%)			
≤1 cm	400 (22.2)	357 (25)	43 (12)
>1 - 2 cm	996 (55.3)	812 (53)	184 (53)
>2 - 3 cm	313 (17.4)	221 (26)	92 (26)
>3	80 (4.4)	51 ()	29 (8)
Unknown	12 (0.7)	10 (<1)	2 (1)
Tumor grade category, *n* (%)			
Grade 1	258 (14.3)	245 (17)	13 (4)
Grade 2	907 (50.4)	742 (51)	165 (47)
Grade 3	297 (16.5)	174 (12)	123 (35)
Not applicable/Unknown	339 (18.8)	290 (20)	49 (14)
Tumor grade and size, *n* (%)			
Grade 1 and tumor size ≤1 cm	90 (5.0)	88 (6)	2 (<1)
Histology, *n* (%)			
IDC	1461 (81.1)	1159 (80)	302 (86)
ILC	213 (11.8)	186 (13)	27 (8)
Mucinous/colloid	53 (2.9)	46 (3)	7 (2)
Papillary	21 (1.2)	19 (1)	2 (1)
Other	53 (2.9)	41 (3)	12 (3)

Abbreviations: IDC, Invasive ductal carcinoma; ILC, invasive lobular carcinoma.

Table S2. Multivariable regression analysis on the entire cohort (chemotherapy treated and untreated) evaluating the association between RS results and clinicopathological characteristics and the presence of distant recurrence; 383 patients were excluded due to missing data on ≥1 covariates.

Variable[a]	Hazard ratio	95% Hazard ratio confidence limits	*P*-value
RS (18-30 vs <18)	2.0	0.97-4.3	< 0.0001
RS (≥31 vs <18)	5.8	2.6-12.9	
Age (per year)	1.0	0.98-1.0	0.976
Size (>2 cm vs ≤2 cm)	2.3	1.3-4.0	0.006
Grade 1 vs 3	0.2	0.02-1.5	0.118
Grade 2 vs 3	1.3	0.7-2.6	

Abbreviations: RS, recurrence Score.

[a] Variables considered in univariate analysis included RS risk group, gender, age, tumor size, tumor grade, and histologic subtype. RS risk group, age, tumor size, and tumor grade were significant prognostic factors in the univariate model.

Figure S1. RS Distribution by Clinical and Pathological Characteristics. RS distribution by age group (**a**), tumor size (**b**), and tumor grade (**c**). Tumor size information was missing for 12 patients; tumor grade information was not available for 339 patients (60% of whom had invasive lobular carcinoma).Green, RS<18; yellow, RS: 18-30; red, RS≥31.

a

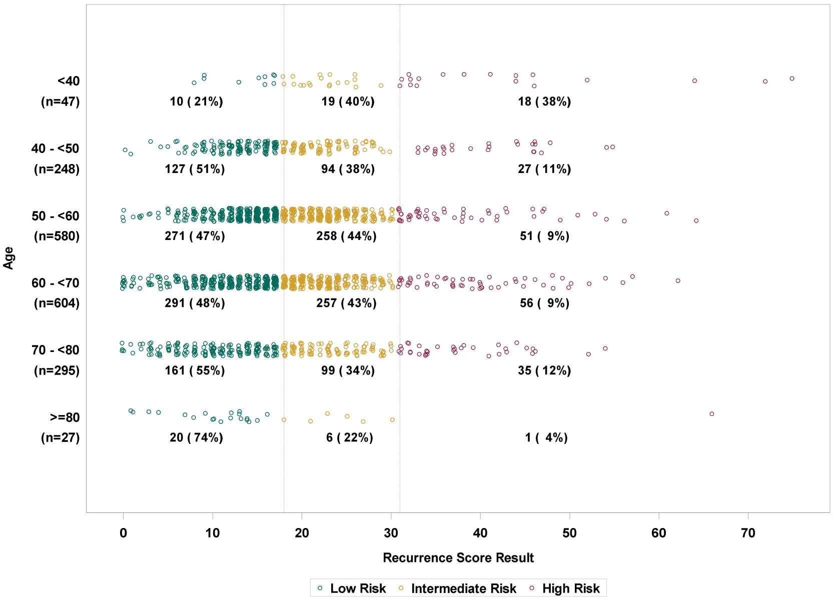

b

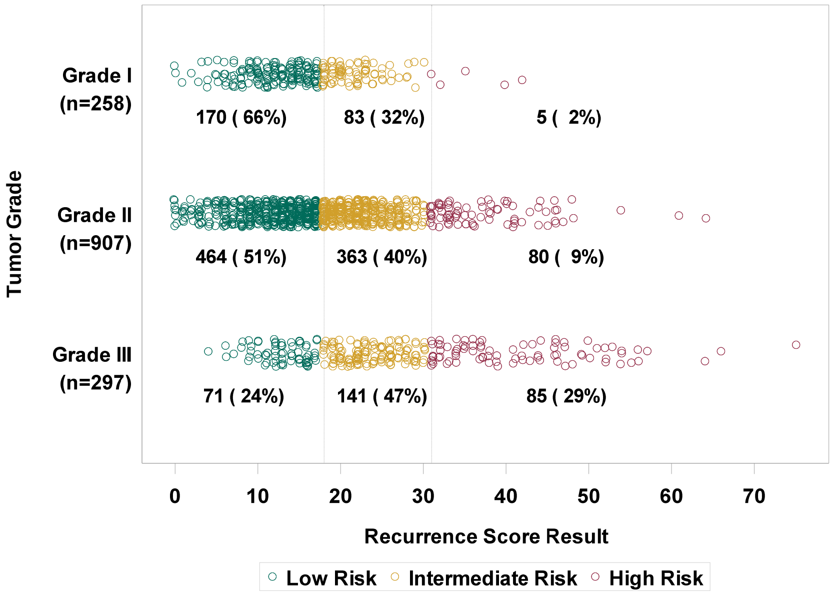

c

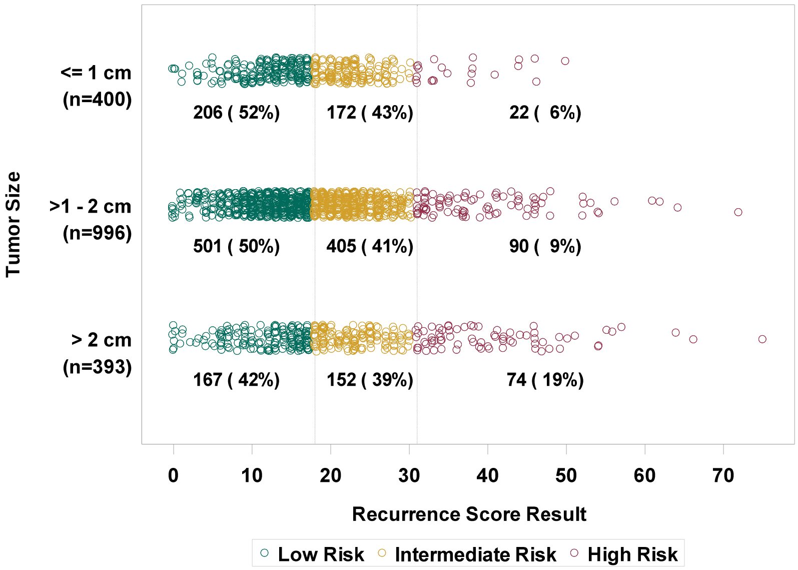
Tumor Size
<= 1 cm
(n=400)
206 (52%)
172 (43%)
22 (6%)
>1 - 2 cm
(n=996)
501 (50%)
405 (41%)
90 (9%)
> 2 cm
(n=393)
167 (42%)
152 (39%)
74 (19%)
0
10
20
30
40
50
60
70
Recurrence Score Result
Low Risk
Intermediate Risk
High Risk

Figure S2. Chemotherapy treatment within patients with RS 18-30 (*n* = 733).

Number of patients per RS result by chemotherapy use is displayed in the bar chart segments.

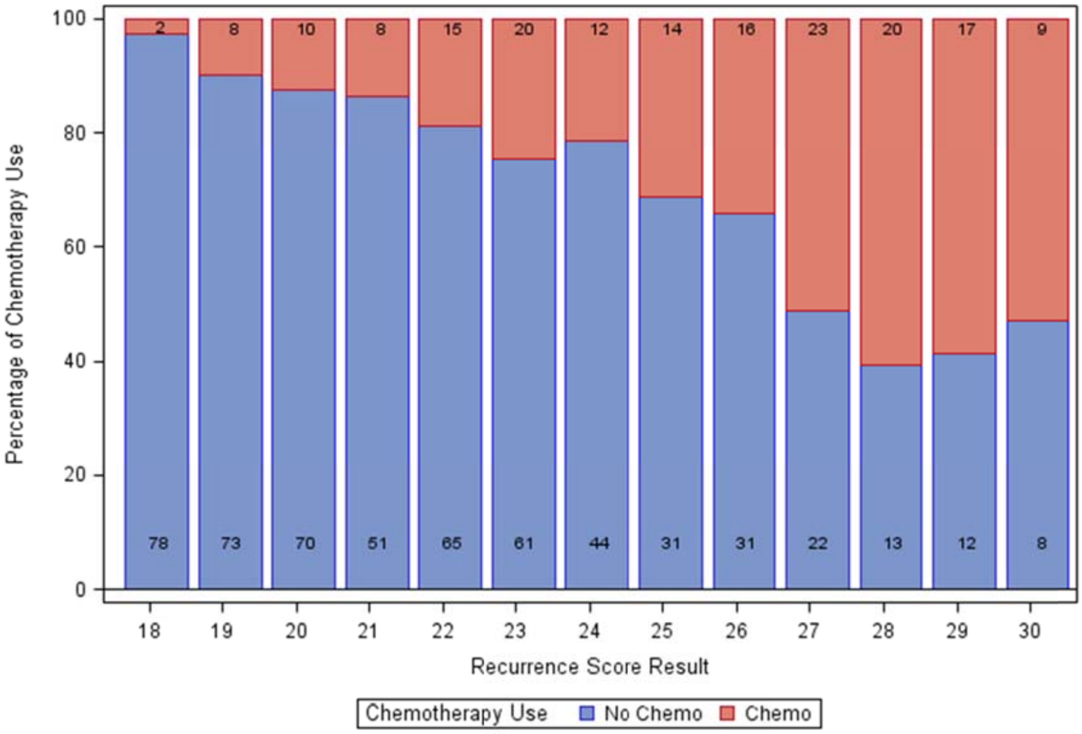

*Number of patients per Recurrence Score Result by chemotherapy use displayed in bar chart segments
